# Noble Metal Organometallic Complexes Display Antiviral Activity against SARS-CoV-2

**DOI:** 10.3390/v13060980

**Published:** 2021-05-25

**Authors:** Christina Chuong, Christine M. DuChane, Emily M. Webb, Pallavi Rai, Jeffrey M. Marano, Chad M. Bernier, Joseph S. Merola, James Weger-Lucarelli

**Affiliations:** 1 Department of Biomedical Sciences and Pathobiology, Virginia Tech, VA-MD Regional College of Veterinary Medicine, Blacksburg, VA 24061, USA; cc6re@vt.edu (C.C.); pallavirai@vt.edu (P.R.); 2 Department of Chemistry, Virginia Tech, Blacksburg, VA 24061, USA; cduchane@vt.edu (C.M.D.); chadb@vt.edu (C.M.B.); 3 Department of Entomology, Virginia Tech, Blacksburg, VA 24061, USA; wemily2@vt.edu; 4 Department of Translational Biology, Medicine, and Health, Virginia Tech, Blacksburg, VA 24061, USA; jmarano@vt.edu

**Keywords:** SARS-CoV-2, COVID-19, organometallics, metallodrugs, antiviral, virucidal

## Abstract

SARS-CoV-2 emerged in 2019 as a devastating viral pathogen with no available preventative or treatment to control what led to the current global pandemic. The continued spread of the virus and increasing death toll necessitate the development of effective antiviral treatments to combat this virus. To this end, we evaluated a new class of organometallic complexes as potential antivirals. Our findings demonstrate that two pentamethylcyclopentadienyl (Cp*) rhodium piano stool complexes, Cp*Rh(1,3-dicyclohexylimidazol-2-ylidene)Cl_2_ (complex 2) and Cp*Rh(dipivaloylmethanato)Cl (complex 4), have direct virucidal activity against SARS-CoV-2. Subsequent in vitro testing suggests that complex 4 is the more stable and effective complex and demonstrates that both 2 and 4 have low toxicity in Vero E6 and Calu-3 cells. The results presented here highlight the potential application of organometallic complexes as antivirals and support further investigation into their activity.

## 1. Introduction

Since the emergence of severe acute respiratory syndrome coronavirus 2 (SARS-CoV-2) in December 2019, approximately 150 million people have been infected worldwide, with approximately a fifth of those cases reported in the USA alone [1,2]. The clinical presentation of the novel coronavirus disease 2019, or COVID-19, commonly includes fever, cough, fatigue, myalgia, headache, and loss of taste and smell. However, more severe disease presenting as pneumonia, acute respiratory distress syndrome (ARDs), septic shock, and multi-organ failure has been responsible for over 2.5 million fatalities [3,4]. Continuous lapses in following social health guidelines combined with the high transmission rate of SARS-CoV-2 have led to several waves of infection and the steady circulation of the virus. Despite recent vaccine development efforts, mass production and realistic distribution to the global public are hindered by time and logistical constraints [5,6]. Additionally, new variants of the virus have challenged the durability of these vaccines for permanent use [7,8,9]. Thus, novel antiviral treatments are critical for controlling the pandemic and decreasing mortality.

In an attempt to respond to the pandemic quickly, there has been a push to repurpose preexisting pharmaceuticals against SARS-CoV-2; however, most have not shown significant benefit. For example, hydroxychloroquine and chloroquine are no longer recommended for use by the National Institutes of Health (NIH) despite early enthusiasm due to poor therapeutic results and side effects contributing to cardiac ventricular arrythmia [10,11]. Other treatments, including HIV protease inhibitors and antiparasitics such as ivermectin, have not been sufficiently studied for use against SARS-CoV-2 [12]. Currently, the nucleoside analogue remdesivir has been the only FDA-approved antiviral for treating COVID-19 [13]. Despite promising results, one study revealed that 24.6% of patients receiving remdesivir had serious adverse effects [14]. In addition to the potentially toxic effects of these drugs, resistance to nucleoside analogues has occurred, thus exposing the limited long-term use of this class of antivirals [15,16]. Therefore, discovering and utilizing antivirals that are not only novel and effective but also resilient against the generation of resistance are critical for addressing the current pandemic and other emerging pathogens.

**Figure 1:**
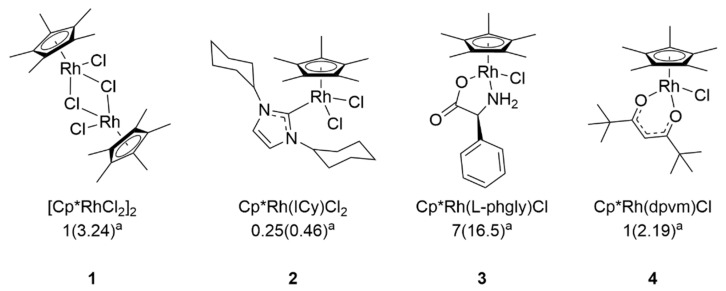
Organometallic complexes investigated for virucidal activity against SARS-CoV-2. ^a^ Minimum inhibitory concentration (MIC) as reported previously and given in µg/mL (µM) against *Mycobacterium smegmatis*, which is commonly used as a model organism for more pathogenic strains of mycobacteria, including *M. tuberculosis* [39]. Cp* = pentamethylcyclopentadienide; ICy = 1,3-dicyclohexylimidazol-2-ylidene; phgly = phenylglycinato; dpvm = dipivaloylmethanato.

Interest in metal-based agents, or metallodrugs, has increased due to the growing rates of antimicrobial and anticancer drug resistance. The efficacy of many classical coordination complexes has been studied against several pathogens, including herpes simplex virus, influenza A virus, SARS-CoV-1, SARS-CoV-2 and others [17,18,19,20]. However, organometallic complexes comprised of noble metals have been shown to be more robust and adaptable drug candidates due to the extensive structural variation and diverse stereochemistry that arise from the incorporation of unique ligand combinations, thus leading to a wide variety of complexes that can be tailored to have a vast range of properties [21,22,23]. While the chemotherapeutic activity of similar complexes has been reported [24,25,26,27,28], as a whole, investigations into the virucidal properties of piano stool complexes are understudied. However, our own studies [29,30,31,32,33] and other reports [22,34,35,36,37,38] have shown that organometallic piano stool complexes have robust antimicrobial activity against highly antibiotic-resistant bacteria including Mycobacterium spp. and Staphylococcus spp. The high antimicrobial activity previously demonstrated by our library of rhodium and iridium piano stool complexes motivated us to evaluate the antiviral activity of a selection of these complexes (Figure 1) against SARS-CoV-2. Our data demonstrated that these organometallics exhibit direct virucidal activity, suggesting that further studies should be conducted to determine their efficacy in vivo or in other applications such as antiviral surface coatings.

## 2. Materials and Methods

### 2.1. Cell and Virus Culture

Vero E6 (CRL-1586) and Calu-3 (HTB-55) cells were obtained from ATCC (Bethesda, MA, USA) and cultured at 37 °C in 5% CO_2_ in Dulbecco’s modified Eagle’s medium (DMEM) supplemented with 5% or 10% fetal bovine serum (FBS), respectively, 1% nonessential amino acids, and 0.1% gentamicin. The SARS-CoV-2 strain utilized was USA-WA1/2020 and obtained from BEI Resources (NR-52281; Lot: 70034262).

### 2.2. Identification and Synthesis of Organometallic Complexes

The complexes utilized in these studies are depicted in Figure 1 and consist of four rhodium complexes, denoted as complex 1–4. Complex 1 is the dimeric rhodium pentamethylcyclopentadienyl (Cp*) chloride, and complexes 2, 3, and 4 are derivatives of this dimer featuring the ligands 1,3-dicyclohexylimidazol-2-ylidene (2), phenylglycinato (3), and dipivaloylmethanato (4). The synthesis of each complex and the corresponding minimum inhibitory concentrations (MIC) against *Mycobacterium smegmatis* have previously been reported [29,31,32,33,40]. Stock solutions of the complexes were formulated at concentrations of 100 μg/mL in 10% DMSO in DI water, and filter-sterilized (0.2 μm) before storage at room temperature.

### 2.3. Direct Virucidal Assays

Diluted complexes were made in RPMI 1640 medium without L-glutamine, supplemented with 10 mM HEPES and 2% FBS (herein called “viral diluent”). The diluted complex solutions were made at 2X concentrations and were inoculated with equal volumes of 150–200 plaque forming units (PFU) of SARS-CoV-2. A 10% DMSO control match was formulated as the highest DMSO concentration present in the least diluted complex. Mixtures were incubated at 37 °C in 5% CO_2_ for 0.5, 1, or 3 h to allow interactions to occur. After the incubation, plaque assays were performed to quantify infectious virus. Confluent 24-well plates of Vero E6 cells were inoculated with 50 μL of the mixtures. After a 1 h adsorption period, 0.5 mL of 1.5% methylcellulose overlay was added to each well and plates were allowed to incubate for 3 days to achieve plaque formation. Plates were then fixed with 10% formalin and stained with crystal violet. Percent plaque reduction was calculated from the number of plaques formed in experimental conditions compared to the number of plaques formed in the DMSO control.

### 2.4. Cytotoxicity Assays

Vero E6 and Calu-3 cells were seeded in 96-well plates one day prior to testing. At 80–90% confluency, growth medium was removed and replaced with medium containing 0.5–50 μg/mL of complex 2 or 4. All treatments were added in quadruplicate or quintuplicate wells. Plates were incubated for 24 h before being washed and replaced with fresh untreated growth medium. MTS was added to each well as recommended by the manufacturer’s protocol (CellTiter 96 Aqueous One Solution Cell Proliferation Assay, Promega, Madison, WI, USA) and incubated for 3–4 h. After incubation, absorbance was measured at 490 nm on a SpectraMax M5 (Molecular Devices, LLC, San Jose, CA, USA) plate reader with SoftMax Pro 6.2.1 software. Viability was calculated by comparing absorbance values to untreated cells and using medium-only wells to control for background absorbance.

### 2.5. Prophylactic and Therapeutic Treatment Antiviral Assays

For prophylactic testing, Vero E6 cells in 24-well plates were used at 90% confluency. Standard growth medium was removed and replaced with viral diluent containing 2.5–25 μg/mL of either complex 2 or 4, or 10% DMSO control. Treated plates were incubated at 37 °C in 5% CO_2_ for 3 h before inoculation with SARS-CoV-2 at a multiplicity of infection (MOI) of 0.1. The virus was allowed to incubate on the cells for 1 h before the wells were gently rinsed twice with PBS. After rinsing, 0.5 mL of growth medium was added to each well and culture supernatant was collected 24 h post infection (hpi). For therapeutic testing, no pre-treatment step was performed. Vero E6 cells in 24-well plates were used at 90% confluency before infecting with SARS-CoV-2 at a MOI of 0.1, as described previously. After rinsing twice with PBS, viral diluent containing 2.5–25 μg/mL of either complex 2 or 4, or 10% DMSO control, was added to each well and allowed to incubate for 3 h at 37 °C in 5% CO_2_. After treatment, cells were rinsed with PBS. Finally, 0.5 mL of growth medium was added to each well and culture supernatant was collected 24 hpi. Plaque assays on the collected supernatants were completed as previously described, except that they were serially diluted 10-fold in viral diluent before being inoculated onto confluent Vero E6 cells. PFUs were counted and multiplied by the dilution factor to determine viral titers per mL of supernatants. Both prophylactic and therapeutic testing were performed in triplicate per dose and control in two biological replicates.

### 2.6. Statistical Analysis

Statistical comparisons were performed in Prism 8 (GraphPad, San Diego, CA, USA). For dose response assays, IC50 values were calculated utilizing nonlinear regression curve fitting. For time dependency assays, simple linear regression and Pearson correlation coefficients tests were conducted. In cytotoxicity, prophylactic and therapeutic assays, samples were compared using 2-way ANOVA with Dunnett’s multiple comparisons test.

## 3. Results

### 3.1. Virucidal Activity of Various Organometallic Complexes

**Figure 2:**
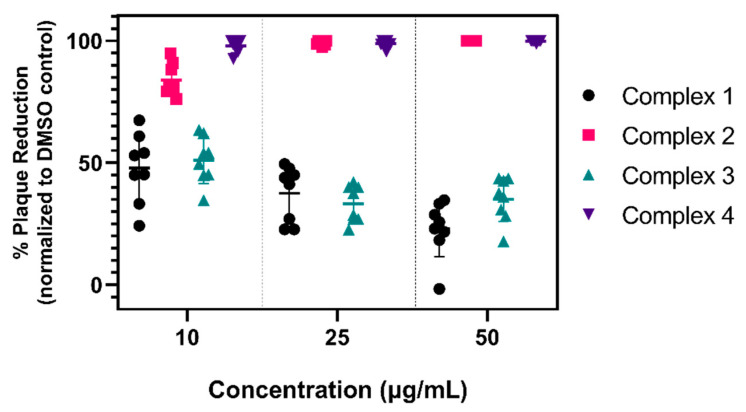
Organometallic complexes exhibit variable antiviral activity against SARS-CoV-2. Each organometallic complex was inoculated with 200 PFU of SARS-CoV-2 at concentrations of 10, 25, and 50 μg/mL and incubated at 37 °C for 3 h. Following incubation, complex–virus mixtures were added to Vero E6 cells for a 1 h adsorption period. Infectious SARS-CoV-2 titers were quantified via plaque assay and data are reported as percent plaque reduction compared to a DMSO–virus control. Data are shown from two biological replicates conducted in quadruplicate per concentration. Horizontal bars represent the mean and error bars represent the standard deviation.

We tested the ability of four candidates (Figure 1) to directly inactivate SARS-CoV-2. Since these types of complexes have not been screened for virucidal activity against SARS-CoV-2, we assessed the activity of the parent dimer (Complex 1), as well as the activities of a variety of piano-stool analogs derived from the dimer (Complexes 2–4) in order to gain a preliminary understanding of structure–activity relationships in these systems. These specific complexes were selected as they showed potent antimycobacterial activity compared to other derivatives based on each structure [29,31,32,33]. A range of concentrations between 10 and 50 μg/mL for each complex were incubated with 200 plaque forming units (PFU) of SARS-CoV-2 for 3 h before inoculation onto Vero E6 cells. The initial incubation time of 3 h was selected based on previous bacteria time–kill measurement assays with similar organometallic complexes [30]. Complexes 2 and 4, which have the lowest MICs against *M. smegmatis*, showed the most robust virucidal activity against SARS-CoV-2 (Figure 2) and were significantly more virucidal than complex 1 and 3 at all concentrations tested (**** p < 0.0001). At 10 μg/mL, complex 2 reduced plaque formation by approximately 84%; in comparison, complex 4 significantly reduced plaque formation by 98% (** p = 0.0021). At concentrations of 25 and 50 μg/mL, complex 2 and 4 resulted in a >99% reduction in SARS-CoV-2. Complex 1 and 3 were less effective at reducing SARS-CoV-2 plaque formation and efficacy did not improve with increasing concentration. These data indicate that the organometallic complexes studied exhibit direct virucidal activity consistent with the complexes that are highly antimicrobial, thus supporting further studies to evaluate these as antiviral drugs.

### 3.2. Organometallic Antiviral Dose–Response

**Figure 3:**
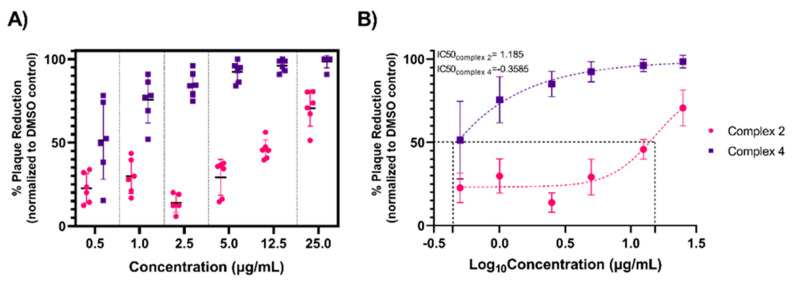
Organometallic Complex 4 shows consistent and robust antiviral activity at low concentrations. (**A**) Complexes 2 and 4 were inoculated with 200 PFU of SARS-CoV-2 at concentrations ranging from 0.5-25 μg/mL and incubated for 3 h at 37 °C to determine the threshold of antiviral activity. Following incubation, complex–virus mixtures were added to Vero E6 cells for a 1 h adsorption period. Infectious SARS-CoV-2 titers were quantified via plaque assay and data are reported as percent plaque reduction compared to a DMSO-virus control. Data were analyzed in (**B**) as a dose-response curve with reported IC50 in Log_10_ Concentration. Data are shown from two biological replicates conducted in triplicate per concentration. Bars represent the mean and error bars represent the standard deviation.

As complexes 2 and 4 exhibited the greatest virucidal activity against SARS-CoV-2, downstream analyses focused on these candidates. We performed a dose–response assay using 0.5–25 μg/mL of each complex to determine the lowest effective virucidal concentration (Figure 3A). Complex 4 consistently reduced infectious SARS-CoV-2 across the gradient, reducing an average of 51.3% of plaques at the lowest concentration, 0.5 μg/mL, while reaching >90% at concentrations starting at 5.0 μg/mL. Surprisingly, complex 2 showed lower efficacy compared to our previous observations (Figure 2). Even at 25 μg/mL, approximately only 70% of infectious virus was reduced. Furthermore, a weaker dose-dependent virucidal effect was observed with complex 2 compared to complex 4. The IC50, defined here as the concentration necessary for 50% inhibition of plaque formation, is shown for both complexes (Figure 3B). The IC50 for complex 4 (Log_10_ Concentration = −0.3585 = 0.4381 μg/mL) is approximately 35-fold greater than complex 2 (Log_10_ Concentration = 1.185 = 15.31 μg/mL). The robust and steady antiviral activity of complex 4 compared to the inconsistent activity of complex 2 possibly indicates the latter may be unstable in solution under biological conditions.

### 3.3. Time Dependence of Antiviral Activity

**Figure 4:**
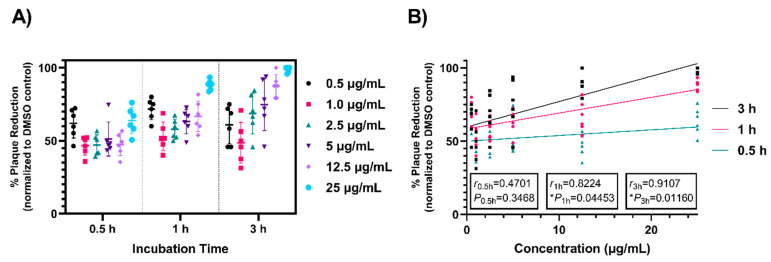
Complex 4 antiviral activity increases with extended incubation periods. (**A**) Complex 4 was inoculated with 150 PFU of SARS-CoV-2 at concentrations ranging from 0.5–25 μg/mL and incubated for 0.5, 1, and 3 h at 37 °C to determine dependency of time on antiviral activity. Following incubation, complex–virus mixtures were added to Vero E6 cells for a 1 h adsorption period. Infectious SARS-CoV-2 titers were quantified via plaque assay and data are reported as percent plaque reduction compared to a DMSO–virus control. Horizonal bars represent the mean and error bars represent the standard deviation. Data were analyzed in (**B**) for correlation of complex 4 concentrations with virus incubation time. r values indicating strength and direction between the two variables and *p* values were determined using Pearson correlation coefficients test. Data are shown from two biological experiments conducted in triplicate. * p = 0.0332, ** p = 0.0021.

Complex 4 displayed significant inactivation of SARS-CoV-2 at low concentrations after 3 h incubation. To analyze the threshold of time needed to achieve virucidal activity, different concentrations of complex 4 were tested at 0.5 and 1 h to compare to 3 h incubation, as previously used (Figure 4A). At concentrations ≥2.5 μg/mL, increased incubation times demonstrated more robust plaque reduction. Maximum virucidal effect was observed with 3 h incubation. Notably, at 0.5 h and 1 h incubation times with 25 μg/mL, SARS-CoV-2 plaque formation was still reduced by 64% and 88%, respectively. Linear regression and correlation analyses determined a positive relationship between complex concentration and incubation time (Figure 4B), with greater strength and direction for increasing incubation times (*r*_0.5h_ = 0.4701, *r*_0.5h_^2^ = 0.2210, *P*_0.5h_ = 0.3468; *r*_1h_ = 0.8224, *r*_1h_^2^ = 0.6763, *P*_1h_ = 0.04453; *r*_3h_ = 0.9107, *r*_3h_^2^ = 0.8294, *P*_3h_ = 0.01160). Incubation times greater than 3 h were not tested as ≥98% plaque reduction was achieved for complex 4 under these conditions. The results presented here reiterate the robust virucidal activity of compound 4 and a time-dependent plaque reducing effect.

### 3.4. Toxicity Testing of Complexes 2 and 4

**Figure 5:**
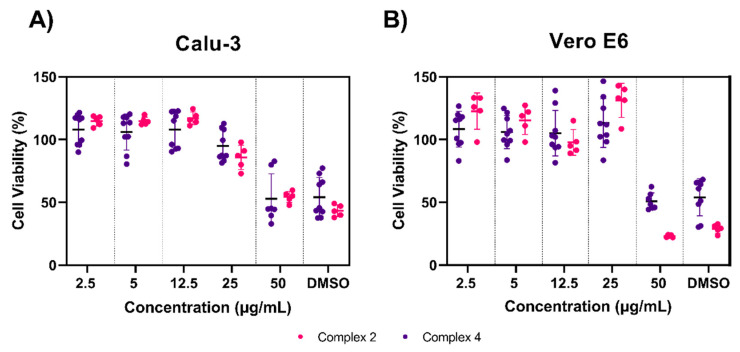
Cytotoxicity of Complexes 2 and 4. Cell viability was measured by MTS assay in (**A**) Calu-3 and (**B**) Vero E6 cell lines. Complex 2 and 4 were made in complete cell medium at concentrations ranging from 2.5–50 μg/mL and allowed to incubate with cells for 24 h before assessing viability. All MTS values were normalized to the control (untreated cells). No statistical differences were observed between concentrations of 50 μg/mL and the DMSO control. All other concentrations were significantly greater than the DMSO controls, **** p < 0.0001. Data are shown from experiments conducted in quadruplicate or quintuplicate wells. Horizontal bars represent the mean and error bars represent the standard deviation.

We next sought to evaluate the safety profile of complexes 2 and 4 at concentrations we previously found to be antiviral using a colorimetric MTS viability assay. We used two cell lines known to be susceptible to SARS-CoV-2 infection: Calu-3 (human lung epithelial cells, Figure 5A) and Vero E6 (African green monkey kidney epithelial cells, Figure 5B). Overall, both complexes were well tolerated in each cell line after 24 h. Considerable cytotoxicity only occurred at the highest concentration tested, 50 μg/mL; at this concentration, in Calu-3 cells, complex 2 decreased cell viability to 54% and complex 4 decreased cell viability to 52% compared to untreated cells; in Vero E6, complex 2 decreased cell viability to 22% and complex 4 decreased cell viability to 50% compared to untreated cells. Of note, these viability drops were comparable to the DMSO controls, suggesting that this toxicity may not be the direct result of the organometallic complexes. Similar concentrations of DMSO have been shown to be cytotoxic to both cell lines in other reports [41,42]. There were no significant differences between 50 μg/mL and DMSO controls for either complex in both cell lines. All other concentrations tested had significantly higher cell viabilities (**** p < 0.0001) compared to the DMSO control, which resulted in >95% cell viability for both cell lines and complexes, with the exception of 25 μg/mL for complex 2 in Calu-3 cells which yielded an 85% cell viability.

### 3.5. Prophylactic and Therapeutic Efficacy of Complexes 2 and 4

**Figure 6:**
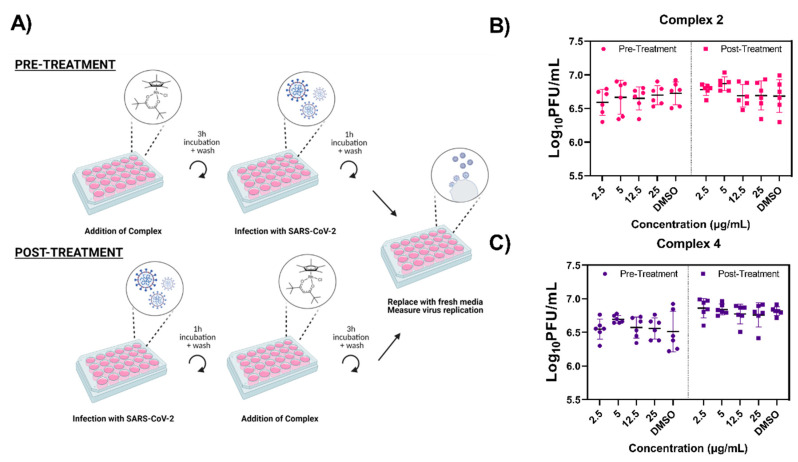
Organometallic complexes lack activity after prophylactic and therapeutic treatment. Vero E6 cells were treated with either complex 2 (**B**) or 4 (**C**) and infected (MOI = 0.1) with SARS-CoV-2 as displayed by the treatment schematic (**A**). Following infection or post-treatment, complete medium was added and supernatant was collected 24 hpi. Infectious SARS-CoV-2 titers were quantified via plaque assay and data are reported as Log_10_ PFU/mL titers. Data are shown from two biological experiments conducted in triplicate. Individual data points are displayed and error bars represent the standard deviation.

To assess the ability of these complexes to alter SARS-CoV-2 replication, we performed a pre-treatment (prophylactic) and post-treatment (therapeutic) assay in Vero E6 cells (Figure 6A). For pre-treatment, the complex or DMSO control was added for 3 h prior to infection with the virus. After infection, the complex was removed and growth medium was added. Culture supernatants were collected at 24 h post-infection (hpi). Viral titers were measured by plaque assay with all samples reaching titers between 6 and 7 Log_10_ PFU/mL (Figure 6B,C). There were no significant differences in viral titer observed in any of the pre-treated cells when compared to the DMSO controls for either complexes.

The therapeutic potential of complex 2 and 4 was examined in a similar manner as described above (Figure 6A). After viral infection of Vero E6 cells, complex 2 and 4 were added directly to infected cells at concentrations ranging from 2.5–25 µg/mL and incubated for 3 h. Following post-treatment, the complex was removed and growth medium added. Culture supernatant was sampled at 24 hpi. As observed with the pre-treatment, all viral titers measured between 6 and 7 Log_10_ PFU/mL (Figure 6B,C) regardless of treatment and no significant differences were observed when compared to the DMSO control. The absence of antiviral activity immediately before and after viral infection suggests that the virucidal mechanism of these complexes may rely on direct contact with the viral particles. Substantial replication of SARS-CoV-2-infected Vero E6 cells begins approximately 8 hpi and gradually increases until plateauing at 14 hpi [43]. Premature removal of the complexes prior to viral budding may explain the lack of antiviral activity observed. However, extended 24 h pre- and post-treatments with complex 4 before and after viral infection also failed to reduce viral titers (Figure S1).

## 4. Discussion

**Figure 7:**
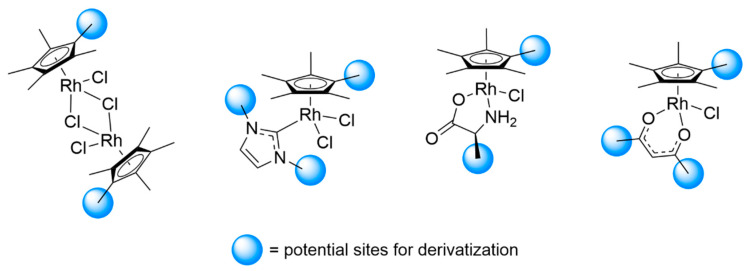
Parent structures of the noble organometallic complexes.

The growing threat of multi-drug-resistant and evolving pathogens requires the development and use of more robust and resilient medications. In this study, we screened several noble metal organometallic complexes for their ability to inactivate the COVID-19 causative agent, SARS-CoV-2. Four initial rhodium candidates were chosen based on their previously characterized antimicrobial activity against *M. smegmatis* [29,31,32,33,40]. We demonstrate that two complexes, Cp*Rh(1,3-dicyclohexylimidazol-2-ylidene)Cl_2_ (complex 2) and Cp*Rh(dipivaloylmethanato)Cl (complex 4), have excellent virucidal activity. Complexes 2 and 4 also had the lowest reported MICs against *M. smegmatis* (0.46 μM and 2.19 μM, respectively). Our work demonstrates that complexes 2 and 4 are able to inactivate SARS-CoV-2 and decrease infectious plaque formation up to 99% on Vero E6 cells in direct virucidal assays. Additionally, increased virucidal activity was observed with extended incubation times up to 3 h. It is possible that incubation times greater than 3 h would have yielded more robust virucidal results at lower concentrations, resulting in lower IC50 values for both complexes. Furthermore, these complexes exhibited low toxicity when tested in Calu-3 and Vero E6 cells. Thus, these candidates serve as excellent examples for safe and effective virucidal applications for noble metal organometallic complexes and provide a framework to further explore other derivations (Figure 7).

The activity of these two compounds may stem from the overall hydrophobicity of the complexes, which are strongly influenced by the ligand environment. Greater overall hydrophobicity has been proposed to facilitate the diffusion of similar complexes through cellular membranes, leading to increased uptake and accumulation of the compounds for anticancer activity [44,45]. Furthermore, naturally occurring [46,47,48] and synthetic peptides [49,50] containing significant hydrophobic regions have shown antiviral activity against enveloped viruses, leading to physical disruption of the viral membrane. It is feasible that similar mechanisms enable complexes 2 and 4 to readily traverse viral envelopes to allow for noble metal-induced antiviral activity. An extension of this study aimed at characterizing the virucidal effect of these complexes on additional enveloped viruses as well as nonenveloped viruses, such as coxsackie B viruses and rotavirus, would aid in affirming this mechanism.

Alternatively, the complexes may act directly on the viral proteins of the virion. Metals such as zinc and copper are common binding partners to several viral proteins [51], and collectively affect the propagation and pathogenesis of viruses including influenza, human coronaviruses, and hepatitis B virus [19,52,53]. Given our robust virucidal results, it is possible that our complexes target the SARS-CoV-2 spike (S) protein, responsible for entry into susceptible host cells. Gold metallodrugs have been shown to bind the S protein of SARS-CoV-2 as one mechanism of antiviral inhibition [54]. Therefore, it is possible that our rhodium organometallic compounds may also bind the S protein, preventing its ability to interact with ACE2, the cellular receptor, or by disrupting fusion, mediated by the S2 subunit of the S protein. Furthermore, the inconsistent antiviral activity with complex 2 and lack of virucidal activity observed in extended prophylactic and therapeutic treatments may indicate a breakdown of these metal–protein interactions. For example, during pre-treatment, the organometallics may enter the cells and the complexes may dissociate before interacting with SARS-CoV-2 virions. In the case of post-treatment, it may be that the complexes are unable to exert organometallic-mediated antiviral activity since the virus has already entered the cells and cannot interact with the S protein. Subsequent uptake of the complexes into the cells post-infection again may promote dissociation and inactivation of rhodium, preventing virucidal activity.

Complex inactivation may stem from halide dissociation over time to give an inactive cationic or dicationic metal complex [55]. Salt supplementation, such as with NaCl or KCl, may serve to deter halide dissociation. Furthermore, the complexes may exhibit sensitivities when suspended in the presence of components in cell culture, primarily bovine serum albumin (BSA). BSA has been documented to interact and bind with metal complexes in other metallodrugs and thus may interfere with antiviral activity [56]. Results were best seen when compounds were immediately resuspended and diluted in minimal medium for direct virucidal assays. Therefore, the sustainable use of these compounds as antivirals in cell culture or in vivo requires investigation into a stable formulation to ensure consistent virucidal activity. Alternatively, these complexes may have utility as antiviral surface coatings. Other metals such as copper, silver, and zinc have been used in conjunction with various polymer coatings in order to make antiviral surfaces [57,58,59], and this may accordingly be a potential practical application for these complexes and should be investigated.

## 5. Conclusions

In summary, this report highlights a new class of antiviral complexes with robust ability to inactivate SARS-CoV-2. Our results support the conclusion that these compounds are non-toxic in several cell lines and are consistent with previous in vivo studies that suggest similar organometallic complexes may have limited toxicity in mammals [56], thus providing support for further biological studies of these complexes. In addition to investigating more stable chemical formulations, antiviral results could be further enhanced by implementing successive doses of these complexes after viral infection. Furthermore, the greater activity of complex 2 and 4 illustrates the high utility of rhodium Cp* complexes by showing that biological activity can be tailored via structural variation of the ligands, which likely affects other properties including stability. Given the efficacy of these noble metal organometallic complexes against SARS-CoV-2, continued studies on these complexes with an array of structural modifications and viruses will provide additional insight into diversifying the application of organometallic complexes as robust antivirals.

## Data Availability

The authors confirm that the data supporting the findings of this study are available within the article and its Supplementary Materials.

## Supplementary Materials

The following are available online at https://www.mdpi.com/article/10.3390/v13060980/s1, Figure S1: Extended pre- and post- treatments with complex 4 do not improve efficacy of prophylactic or therapeutic activity.
