# Supplementary material for: Noble Metal Organometallic Complexes Display Antiviral Activity against SARS-CoV-2
Source: Viruses. 2021 May 25;13(6):980. doi: 10.3390/v13060980 (PMC8227008; doi:10.3390/v13060980)
Supplement: Supplementary file 1 [file viruses-13-00980-s001.zip › viruses-1218503-supplementary.pdf]

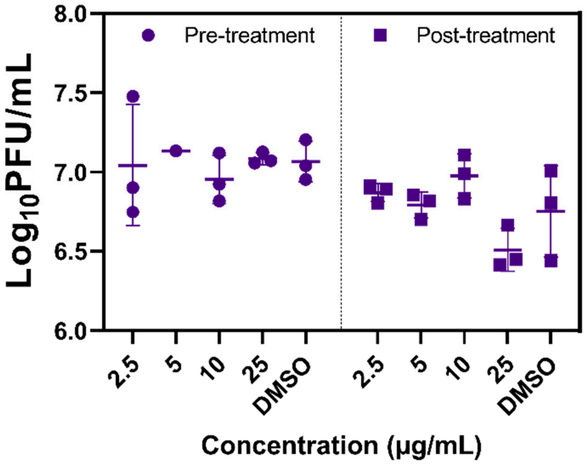

Supplementary Figure S1. Extended pre- and post- treatments with complex 4 do not improve efficacy of prophylactic or therapeutic activity. Vero E6 cells were infected (MOI=0.1) with SARS-CoV-2 after pre-treatment or before post-treatment of cells with complex 4 for 24 h to increase the threshold of prophalytic and therapeutic antiviral activity. Following infection or post-treatment, complete medium was added and supernatant was collected 24 hpi. Infectious SARS-CoV-2 titers were quantified via plaque assay and data are reported as log_{10}PFU/mL titers. Data are shown from one biological experiment conducted in triplicate. Individual data points are shown with error bars representing standard deviation.
